# Supplementary material for: Machine learning image analysis predicts Parkinson disease genotype and mitochondrial lysosomal abnormalities in iPS neurons
Source: NPJ Parkinsons Dis. 2026 Aug 1;12:255. doi: 10.1038/s41531-026-01458-w (PMC13680355; doi:10.1038/s41531-026-01458-w)
Supplement: Supplementary file 1 [file 41531_2026_1458_MOESM1_ESM.docx]

**Supplementary figures**

**Supplementary figure 1. Representative mitochondrial and lysosomal images across control and Parkinson’s disease genotypes.** Representative fluorescence images of iPS-derived neurons from control, *LRRK2*, *PRKN*, and *SNCA* groups labelled with MitoTracker (red) to visualise mitochondria and LysoTracker (yellow) to visualise lysosomes, with merged images including nuclear staining (blue).

**Supplementary figure 2. Cortical neuron characterization across control and Parkinson’s disease genotypes.** Representative immunofluorescence images showing expression of (A) the layer VI cortical marker (A) TBR1 (yellow) and (B) layer V cortical marker CTIP2 (yellow) with Hoechst nuclear staining (blue) and the neuronal marker MAP2 (green), alongside quantification of the percentage of (A) TBR1 and (B) CTIP2 positive cells in control, *LRRK2*, *PRKN*, and *SNCA* iPSC-derived neurons. Data are shown as mean ± SEM.

**Supplementary figure 3. Segmentation workflow used to define cellular regions for morphomic feature extraction. (A)** Representative input image showing nuclei labelled with Hoechst and the cellular compartment labelled with Cytopainter Green dye. These channels were used to guide nuclear and whole-cell segmentation. Scale bar = 100 µm. **(B)** Single-cell segmentation output showing individual cell masks generated from the input image. **(C)** Representative segmented cell showing the five regions used for morphomic feature extraction: four concentric sub-regions (nuclear, inner, middle, and outer) and the whole-cell region, which encompasses all sub-regions. The nuclear region was defined from the Hoechst nuclear mask and the whole-cell region from the Cytopainter dye-derived cell mask; the inner, middle, and outer sub-regions were generated as concentric compartments at increasing radial distance between the nucleus and the cell boundary, with the outer region representing the cell area most distal to the nucleus. Region-specific intensity, texture, morphology, and spatial features were then extracted from the mitochondrial and lysosomal channels within each region.

**Supplementary figure 4. Generalisability of the morphomics machine-learning framework across unseen cell lines.** (A) Leave-one-cell-line-out cross-validation (LOOCV) of the Control vs PD classifier showing mean prediction probabilities for control and PD cell lines. Each datapoint represents the mean prediction probability per cell line, with boxplots indicating median and interquartile range. The model maintains separation between Control and PD groups across unseen cell lines. Colours indicate cell type per cell line. (B) Receiver operating characteristic (ROC) curve for classification of Control vs PD. The model achieved an AUC of 0.81 from a PD vs Control classifier (n = 9 PD cell lines, n = 4 control cell lines).

**Supplementary tables**

| **Antibody** | **Company** | **Catalogue no** | **Species** | **Dilution** |
| --- | --- | --- | --- | --- |
| TBR1 | Abcam | ab31940 | rabbit | 1:1000 |
| CTIP2 | Abcam | ab18465 | rat | 1:1000 |
| MAP2 | Abcam | ab254143 | mouse | 1:1000 |

**Supplementary Table 1. Antibodies. Details for primary antibodies employed for immunocytochemistry.**

| **Antibody** | **Company** | **Catalogue no** | **Species** | **Dilution** |
| --- | --- | --- | --- | --- |
| Goat anti-mouse-488 | Invitrogen | A-11029 | Goat | 1:500 |
| Goat anti-rabbit-488 | Invitrogen | A-11008 | Goat | 1:500 |
| Goat anti-rabbit-594 | Invitrogen | A-11012 | Goat | 1:500 |
| Goat anti-rat-647 | Invitrogen | A-48272 | Goat | 1:500 |
| Goat anti-rat-488 | Invitrogen | A-11006 | Goat | 1:500 |

**Supplementary Table 2. Secondary Antibodies. Details for secondary antibodies employed for immunocytochemistry.**

| **Serial no** | **Assay** | **Stain / reagent** | **Company** | **Catalogue no** | **Final concentration** |
| --- | --- | --- | --- | --- | --- |
| 1 | Mitochondrial TMRM assay (Figure 4) | Tetramethylrhodamine, Methyl Ester, Perchlorate (TMRM) | Invitrogen | T668 | 25 nM |
| 2 |  | Calcein | Invitrogen | C3100 | 500 nM |
| 3 |  | Hoechst | Thermo Scientific | 62249 | 1 µg/ml |
| 4 | Mitotracker and Lysotracker assay (Figures 1-4) | Mitotracker Deep Red | Thermo Fisher | M22426 | 100 nM |
| 5 |  | Cytopainter green cytoplasm marker | Abcam | ab138891 | 1x |
| 6 |  | Lysosomal staining reagent – orange- cytopainter | Abcam | ab176827 | 1x |
| 7 |  | Pureblu Hoechst 33342 | Bio-Rad | 1351304 | 2 µM |

**Supplementary Table 3**. Live cell imaging dyes used for mitochondria and lysosome live cell assay.

| **Assay** | **Lab 1 or 2** | **Number of batches (plates)** | **Objective** | **Number of wells per plate per cell line** | **Z-stack: Number of planes** | **Z-stack: Step size** | **Number of fields of view imaged per well** | **Channel number (pre-defined filter)** | **Imaged** |
| --- | --- | --- | --- | --- | --- | --- | --- | --- | --- |
| Mitochondria and lysosome assay | Lab 1 | 3 | 40x | 3 | 3 | 0.5µm | 25 | Ch1 (Lysotracker Red DND) | Lysosomes |
|  |  |  |  |  |  |  |  | Ch2 (Mitoctracker DeepRed) | Mitochondria |
|  |  |  |  |  |  |  |  | Ch3 (Cytopainter) | Cytoplasm |
|  |  |  |  |  |  |  |  | Ch4 (Hoechst) | Nuclei |
|  |  |  | 40x | 3 | 3 | 0.5µm | 25 | Ch1 (Mitotracker Orange) | Lysosomes |
|  |  |  |  |  |  |  |  | Ch2 (Mitoctracker DeepRed) | Mitochondria |
|  |  |  |  |  |  |  |  | Ch3 (Cytopainter) | Cytoplasm |
|  |  |  |  |  |  |  |  | Ch4 (Hoechst) | Nuclei |
|  |  |  | 40x | 3 | 4 | 0.5µm | 25 | Ch1 (Lysotracker Orange DND) | Lysosomes |
|  |  |  |  |  |  |  |  | Ch2 (Mitoctracker DeepRed) | Mitochondria |
|  |  |  |  |  |  |  |  | Ch3 (Cytopainter) | Cytoplasm |
|  |  |  |  |  |  |  |  | Ch4 (Hoechst) | Nuclei |
| Mitochondria (TMRM) assay | Lab 2 | 3 | 20x | 3 | 3 | 2µm | 15 | Ch1 (Alexa 488) | Cytoplasm |
|  |  |  |  |  |  |  |  | Ch2 (Alexa 568) | Mitochondria (TMRM) |
|  |  |  |  |  |  |  |  | Ch3 (CellMask DeepRed) | Mitochondria (Mitotracker) |
|  |  |  |  |  |  |  |  | Ch4 (Hoechst) 33342 | Nuclei |
|  |  |  | 20x | 3 | 3 | 2µm | 15 | Ch1 (Alexa 488) | Cytoplasm |
|  |  |  |  |  |  |  |  | Ch2 (Alexa 568) | Mitochondria (TMRM) |
|  |  |  |  |  |  |  |  | Ch3 (Brightfield) | Brightfield |
|  |  |  |  |  |  |  |  | Ch4 (Digital Phase Contrast) | Digital Phase Contrast |
|  |  |  |  |  |  |  |  | Ch5 (Hoechst 33342) | Nuclei |
|  |  |  | 20x | 3 | 3 | 2µm | 15 | Ch1 (Alexa 488) | Cytoplasm |
|  |  |  |  |  |  |  |  | Ch2 (Alexa 568) | Mitochondria (TMRM) |
|  |  |  |  |  |  |  |  | Ch3 (Brightfield) | Brightfield |
|  |  |  |  |  |  |  |  | Ch4 (Digital Phase Contrast) | Digital Phase Contrast |
|  |  |  |  |  |  |  |  | Ch5 (Hoechst 33342) | Nuclei |

**Supplementary Table4**. Imaging acquisition parameters for mitochondrial and lysosomal assays.

|  | Training | Validation | Test |
| --- | --- | --- | --- |
| **PD vs Controls** |  |  |  |
| PD | 12560 | 7802 | 7803 |
| Controls | 12560 | 4187 | 4187 |
| **PRKN vs Controls** |  |  |  |
| Controls | 10504 | 4187 | 4187 |
| *PRKN* | 10504 | 3501 | 3501 |
| ***LRRK2* vs Controls** |  |  |  |
| *LRRK2* | 2365 | 788 | 788 |
| Controls | 2365 | 4187 | 4187 |
| ***SNCA* vs Controls** |  |  |  |
| *SNCA* | 10539 | 3513 | 3514 |
| Controls | 10539 | 4187 | 4187 |
| ***PRKN* vs *LRRK2* vs *SNCA*** |  |  |  |
| *PRKN* | 2365 | 3501 | 3501 |
| *LRRK2* | 2365 | 788 | 788 |
| *SNCA* | 2365 | 3513 | 3514 |
| ***PRKN* vs *LRRK2* vs *SNCA* (Laboratory 2)** |  |  |  |
| *PRKN* | 7407 | 5972 | 5972 |
| *LRRK2* | 7407 | 2469 | 2469 |
| *SNCA* | 7407 | 7919 | 7920 |

**Supplementary Table 5**. Cell counts used for training, validation, and test datasets for each classification model.
